# Effective biomedical document classification for identifying publications relevant to the mouse Gene Expression Database (GXD)

**DOI:** 10.1093/database/bax017

**Published:** 2017-03-24

**Authors:** Xiangying Jiang, Martin Ringwald, Judith Blake, Hagit Shatkay

**Affiliations:** 1 Department of Computer and Information Sciences, University of Delaware, 101 Smith Hall, Newark, DE, USA; 2 Department of Computer and Information Sciences, The Jackson Laboratory, 600 Main Street, Bar Harbor, ME, USA

## Abstract

The Gene Expression Database (GXD) is a comprehensive online database within the Mouse Genome Informatics resource, aiming to provide available information about endogenous gene expression during mouse development. The information stems primarily from many thousands of biomedical publications that database curators must go through and read. Given the very large number of biomedical papers published each year, automatic document classification plays an important role in biomedical research. Specifically, an effective and efficient document classifier is needed for supporting the GXD annotation workflow. We present here an effective yet relatively simple classification scheme, which uses readily available tools while employing feature selection, aiming to assist curators in identifying publications relevant to GXD. We examine the performance of our method over a large manually curated dataset, consisting of more than 25 000 PubMed abstracts, of which about half are curated as relevant to GXD while the other half as irrelevant to GXD. In addition to text from title-and-abstract, we also consider image captions, an important information source that we integrate into our method. We apply a captions-based classifier to a subset of about 3300 documents, for which the full text of the curated articles is available. The results demonstrate that our proposed approach is robust and effectively addresses the GXD document classification. Moreover, using information obtained from image captions clearly improves performance, compared to title and abstract alone, affirming the utility of image captions as a substantial evidence source for automatically determining the relevance of biomedical publications to a specific subject area.

**Database URL:** www.informatics.jax.org

## Introduction

Automatically collecting and searching biomedical publications plays an important role in biomedical research, because much information is conveyed in the form of publications. However, since the number of biomedical publications has grown rapidly over the past few decades (1), it becomes impractical for researchers to quickly find all and only those biomedical publications that are related to their needs. One way to address this challenge is through automatic categorization of large amounts of publications by relevance to a topic, which can potentially save considerable time and resources. As such, automated biomedical document classification, aiming to identify publications relevant to a specific research field, is an important task that has attracted much interest (2–7).

The Mouse Genome Informatics (MGI; http://www.informatics.jax.org/) database is the most comprehensive international resource focused on the laboratory mouse as a model organism, providing integrated genetic, genomic and biological data for facilitating study of human health and disease. MGI covers several databases, namely the Mouse Genome Database (8), the Gene Expression Database (9, 10), the Mouse Tumor Biology Database (11) and the MouseMine project (12). The Gene Expression Database (GXD), on which we focus here, is an extensive resource of mouse development expression information. GXD collects and integrates data from a wide variety of expression experiments (including both RNA and protein). Expression data from wild-type and mutant mice are captured, with a focus on endogenous gene expression during development. Knock-in reporter studies are included because they usually reflect the endogenous expression pattern of the targeted gene. However, studies reporting on ectopic gene expression via the use of transgenes, or experiments studying the effects of treatments or other external/environmental factors are excluded.

Much of the detailed information provided by GXD is manually curated from the literature. Among all the publications surveyed MGI-wide, GXD curators first identify those that meet the criteria described above.

Once the publications are identified, the curators annotate within them the genes and ages analyzed and the types of expression assays used. These annotations are combined with the publications information from PubMed to create a searchable index of published experiments on endogenous gene expression during mouse development (http://www.informatics.jax.org/gxdlit). This index allows researchers to find publications with specific types of expression data. Moreover, it supports GXD staff in prioritizing papers toward a more extensive curation step, namely, the detailed annotation of the expression results. The comprehensive up-to-date index, includes Expression Literature Content Records for >24 000 publications and over 15 000 genes.

In the work presented here we use this large and well-curated dataset to train and test a classifier that partitions publications in MGI into those that are relevant for GXD and those that are not. This classification task, referred to as *triage*, is important toward expediting literature curation of developmental expression information.

Basic mainstream methods for biomedical document classification typically use information obtained from titles and abstracts of publications. Lakiotaki *et al.* (4) presented a method for classifying a small manually tagged subset (474 articles) of the OHSUMED TREC collection (13) into documents aimed for medical professionals (clinicians) and those aimed at consumers by employing a medical document indexing method, AMTEx (14). The latter uses MeSH (15) as a basis for vector representation of medical documents and for classification. The system obtained high precision (94.49%) but low recall (50.85%). Ren *et al.* (16) also used vector representation, reduced via feature selection, for identifying 9 different experimental designs in the context of neuroimaging, within a small set of 247 published abstracts from human neuroimaging journal articles selected from BrainMap (17, 18). The reported level of performance using precision and recall were 40 and 85% on average, respectively. Notably, these datasets are both more than an order-of-magnitude smaller than the number of documents considered by GXD, and the tasks addressed do not reflect the magnitude and complexity of the GXD classification challenge.

Larger scale experiments are reported by Yu *et al.* (19), who developed the TopicalMeSH representation, using MeSH terms as a basis for latent topic vectors, to classify a set of 18 000 drug review documents as *relevant* or *irrelevant* to each of 15 classes based on specific treatment conditions. The reported performance, as measured by the *F-measure* (combining precision and recall), was about 50% per class. Aside from the relatively low level of performance, as MeSH terms are assigned to articles by the NLM only several months after publication, a classification system that relies on MeSH annotations or on AMTEx cannot be applied to new publications that have not yet been assigned MeSH terms. As GXD directly examines new publications as soon as they are available, a classification system relying on MeSH annotations is not an effective route to pursue.

Another large scale work by Van Auken *et al.* (6) is described in the context of the Textpresso information retrieval system (20), identifying documents relevant to the WarmBase database (21, 22). They employ support vector machines (SVMs) as a basis for a semi-automated curation workflow. However, the reported classification results are described for sentences rather than for abstracts or papers, and the reported level of performance is on average *50% recall, 80% precision* and *60% F-measure*. The same group also reported applying Textpresso to a small set of documents, containing 55 research articles, to identify relevant papers for Gene Ontology Cellular Component curation (23), obtaining 79.1% *recall* and 61.8% *precision* (7).

In contrast to classification systems that use term-frequency-based document representation, Rinaldi *et al.* (5) introduced the OntoGene system (24), relying on advanced natural language processing (NLP) tools to support rich semantic document representation. The OntoGene system was used for the triage task of BioCreative’12 (25) aiming to select and prioritize documents within a relatively small dataset of 1,725 publications relevant to Comparative Toxicogennomics The system ranks publications by relevance, based on the extraction of target entities and of interactions in which the target entities are involved. OntoGene’s reported performance, which was the best among the participants in the task, is about 80% precision and recall. The method has not been shown sufficiently fast to be applicable in practice, and has not been applied to a large dataset of GXD’s magnitude. To summarize, while some of the existing methods perform effectively on a small-scale specific task, they have not shown to be a good fit for the large-scale GXD categorization task with which we are concerned.

Another important distinction between previous work and the work presented here is that the above methods use text obtained only from PubMed abstracts or drug reviews. However, image captions in the biomedical literatures often contain significant and useful information for determining the topic discussed in the publications, as our group and sevearl others have noted before (26–29). As such, we consider here the use of text obtained from image captions as part of the GXD classification process.

We present a biomedical document classification scheme that uses statistical feature selection to reduce the representation size and to focus the representation on terms that support the GXD classification task. The classifiers we employ and compare are widely applied in biomedical document classification, namely Naïve Bayes (30) and Random Forest classifiers (31–33). Our experiments and results, performed over a set of many thousands of documents, demonstrate that using these relatively simple classifiers, coupled with a well-targetted feature-selection method, lead to highly accurate and stable classification. The importance of feature selection was also shown by others before (34, 35). Our system retains its high level of performance even when applied to the very large set of documents considered by GXD. We also provide results obtained from integrating image captions into the classification process, showing an improved performance. The latter demonstrates the utility and importance of using image captions for supporting biomedical document classification.

## Materials and methods

### Data source

We downloaded from the MGI references website (http://www.informatics.jax.org/reference/) a file that contains information pertaining to all 115 027 publications used to curate gene expression information for the MGI database throughout the years 2004–2014. The information includes: PubMed identifier (PMID), title, publication year and a curated indicator stating the sub-project within MGI to which the correponding paper is relevant. From the references included in the file, we identified 13 035 PMIDs of publications that have the indication *Expression Literature Records* or *Expression: Assay Results* shown in the *Curated Data* column. The topics discussed in these publications are considered *relevant* to GXD. Of these 13 035 *relevant* publications, only 12 966 were available for free download online in PDF format, and we use those and refer to them as the *positive* set in our experiments. Of the remaining 101 992 publications in the downloaded references file after the above selection process, 79 284 publications were available for free download online in PDF format. These publications are considered unrelated or *irrelevant* to GXD. Notably, there is a significant imbalance between the number of available *relevant* examples (12 966) and that of *irrelevant* examples (79 284). It is important to directly address the imbalance in order to train a stable, sensitive and specific classifier under such an imbalanced setting (36). To avoid over-simplification of the classification task (e.g. hypothetically—simply classifying all documents as *irrelevant* would already yield a rather high accuracy), we select 12 354 publications out of the 79 284 *irrelevant* publications to comprise the actual *negative set*. In order to overcome potential bias stemming from varied language use and topic distribution differences between the *positive* set and the *negative* set, the publications included in the *negative* set are obtained from the same journals as the *relevant* ones, and have a similar distribution of publication-years as the *rele*vant ones. The 12 354 *negative* publications are thus selected at random from among the *irrelavant* publications that satisfy the two conditions. By keeping the size of the *negative* set similar to that of the *positive set* and avoiding shift in time, we ensure similarity of writing style and overall areas of interest between the *positive* set and the *negative* set, and avoid the issue of semantic drift between the two sets; a phenomenon that may otherwise bias classification (37).

Image captions, which provide descriptions for figures, form an important source of information in publications. To integrate information from image captions into the classifier, we build an additional datatset, to which we refer as the *GXD-caption* dataset, in which the text associated with each publication consists not only of title and abstract but also of image captions within the paper. Of the 12 966 publications in our *positive* set, 1,630 were availabe in plain text format from the PMC Author Manuscript Collection (38) allowing us to easily obtain figure captions. These documents, for which we have access to titles, abstracts and image captions comprise the *positive GXD-caption* set. Among the 79 284 *irrelevant* publications, 11 099 papers were available in plain text format from the PMC, from which figure captions can be readily obtained. As described before, to retain balance, we select 1,696 out of these 11 099 publications to bulid the *negative GXD-caption* set, similar in size to the *positive GXD-caption set.* The publications in the *negative GXD-caption set* are selected at random from a set of papers published in the same journals and having a similar distribution of publication-years as the publications in the *positive GXD-caption set*. We train/test our classifiers over the combined set including both *positive* and *negative* documents. Table 1 summarizes the datasets used in our experiments.

**Table 1.** The datasets used for training and testing of our biomedical document classification

Dataset	Number of examples		
	Positive	Negative	Total
GXD	12 966	12 354	25 320
GXD-caption	1630	1696	3326

As an additional verification step, we also test our classifiers over an additional subset of 1,000 *irrelevant* documents outside the training/test set. These documents were selected uniformly at random from among the remaining 9,403 *irrelevant* documents for which figure captions are available.

### Document representation

Our document representation is based on a variation on the bag-of-terms model that we have introduced and used in our earlier work (39, 40, 41). The representation uses a set of terms consisting of both unigrams (single words) and bigrams (pairs of two consecutive words). Using a limited number of meaningful terms as features has proven effective and efficient in our earlier work. To reduce the number of features, we first remove standard stop words (42); we also remove rare terms (appearing in a single publication within the dataset), as well as overly frequent ones (appearing within over 60% of the publications in the dataset). The last dimensionality-reduction step, which we introduced before (39), employs the Z-score Test (43) to select features whose probability to occur in the *positive set* is statistically significantly different from their probability to occur in the *negative* set. The Z-score calculation process is described next.

Let *t* be a term, *d* be a publication, *D_r_* denote the set of GXD-*relevant* documents and *D_n_* denote the set of documents irrelevant to GXD. The probability of term *t* to occur within *relevant* publications, *Pr(t|D_r_)*, is estimated as:

$$\mathrm{Pr}\left(t||D_{r}\right)\approx \frac{\#\;\mathrm{of}\;\mathrm{documents}\;\mathrm{in}\;D_{r}\;\mathrm{that}\;\mathrm{have}\;\mathrm{term}\;t}{\mathrm{Total}\;\#\;\mathrm{of}\;\mathrm{documents}\;\mathrm{in}\;D_{r}}\;.$$

Similarly, the probability of term *t* to occur within *irrelevant* publications, *Pr(t|D_n_)*, is estimated as:

$$\mathrm{Pr}\left(t||D_{n}\right)\approx \frac{\#\;\mathrm{of}\;\mathrm{documents}\;\mathrm{in}\;D_{n}\;\mathrm{that}\;\mathrm{have}\;\mathrm{term}\;t}{\mathrm{Total}\;\#\;\mathrm{of}\;\mathrm{documents}\;\mathrm{in}\;D_{n}}\;.$$

We calculate *Pr(t|D_r_)* and *Pr(t|D_n_)* for each term *t*.

Using the formulation above, a term *t* is considered *distinguishing* for the Gene Expression topic, if and only if its probability to occur in publications associated with GXD, *Pr(t|D_r_)*, is statistically significantly different from its probability to occur in publications not associated with GXD, *Pr(t|D_n_)*. To determine the significance of the difference between these two probabilities, the Z-score statistic is employed (39, 43), where:

$$Z\_\mathrm{score}=\frac{\mathrm{Pr}\left(t||D_{r}\right)-\mathrm{Pr}\left(t||D_{n}\right)}{\sqrt{\overset{-}{p}\left(1-\overset{-}{p}\right)\left(\frac{1}{\left|D_{r}\right|}+\frac{1}{\left|D_{n}\right|}\right)}}\;\;,$$

and

$$\overset{-}{p}=\frac{\left|D_{r}\right|\times \mathrm{Pr}\left(t|D_{r}\right)+|D_{n}|\times Pr(t|D_{n})}{\left|D_{r}\right|+|D_{n}|}$$

The higher the absolute value of the Z-score, the greater the confidence level that the difference between *Pr(t|D_r_)* and *Pr(t|D_n_)* is significant. For the publications pertaining to the *GXD* dataset that we have constructed, we set a threshold of 1.96 for the Z-score, that is, if the Z-score of a term is higher than 1.96, it is considered to be *distinguishing* with respect to our classification task. For the documents in the *GXD-caption* dataset, we employ two different Z-score thresholds: one for selecting *distinguishing* terms from the captions, set at 1.63, and one for selecting terms from the titles/abstracts, set at 1.96.

To ensure that the test set is not used for feature selection and is excluded from both the representation and the classification-learning process, the feature selection steps discussed above use only data from the training set. After *n distinguishing* terms are identified through the feature selection process from the training set, these *distinguishing* terms are used to represent the documents in the test set. Each document *d* in the test set is represented as a simple binary vector of the form *V_d_ = <vt_1_, vt_2_, …, vt_n_>*, where *vt_i_=1* if the *i^th^* term in the *distinguishing* terms list is presented in article *d*, and 0 otherwise.

### Classifiers

We trained and tested two types of classifiers, Naïve Bayes and Random Forest, over the *GXD* dataset and over the *GXD-caption* dataset, based on the document representation desribed in the *Document Representaiton* part. The Weka implementation was used to train and test the classifiers (44).

The first classifier used is the Naïve Bayes, which is a simple probabilistic classifier based on the assumption that the value of each feature is conditionally independent of all other features, given the class value. In our case, to determine if a publication *d* is relevant to GXD, the posterior probability *P(d|relevant)* is compared to the posterior probability *P(d|irrelevant)*. If *P(d|relevant)* is greater, publication *d* is classified as *relevant*, otherwise, it is labeled as *irrelevant*. As the Naïve Bayes classifier is simple and fast, it is readily applicable to a large and high-dimensional dataset and we use it in our experiments.

We also ran experiments using the Random Forest classifier. The Random Forest consists of an ensemble of tree-structure classifiers, such that each node in each tree checks for the value of a subset of features, typically chosen through a stochastic process called ‘feature bagging’ (32). If one or a few of the features are strong predictors for the target class, these features are checked in many of the trees, causing the trees to become correlated (33). The Random Forest classifier has shown to be applicable to high-dimensional high-volume data. We thus use it for the GXD document classification task. The forest consists of 2000 decision trees, and the number of features stochastically selected for each tree is set to 90.

## Experiments and results

### Experiments

We first conducted experiments over the large *GXD* dataset, in wich each document is represented based on title-and-abstract only. We then conducted experiments over the *GXD-caption* dataset, in which captions are also included in the representation. In addition, we also tested our classifier that uses title, abstract and captions over a set of 1,000 *irrelevant* documents selected as described in the Methods section.

The first group of experiments aims to test the performance of the proposed classification method over the large scale *GXD* dataset. To ensure our results are statistically significant, we trained/tested the classifiers using *three sets* of cross-validation runs with different settings, as described next.

For the first set of cross-validation experiments over the *GXD* dataset, to ensure stability of the results, we executed five distinct complete 5-fold cross validation runs, each run using a different 5-way split of the dataset, so that each complete run consists of 25 training/test sessions in total. In each session, 80% of the data was used for the training process, in which about 11 400 *distinguishing* terms were selected to repersent publications and classifiers were trained based on the represented publications, while 20% of the data was used for testing the classifiers.

To validate that the classification results remain steady even when the size of training set varies, we performed two additional sets of experiments. In one (to which we refer as the *Second Set* of experiments over *GXD* dataset), we enlarged the size of the training set to be 90% of the data, while reducing the test size to 10%, by running 10-fold cross validation. As before, we employed 10 complete sets of stratified 10-fold cross validation, so that each complete run consists of 100 training/test sessions in total. In each session, about 12 500 *distinguishing* terms were selected from the training part of the data and were used to represent documents in the test set.

In the third set of experiments, we reduced the size of the training set, and increased the size of the test set, by dividing both the *positive* set and the *negative* set into just two subsets: half of the data was treated as the training set while the other half became the test set. The subsets were formed so that the training and the test sets have a similar distribution of publication-years. We trained the Random Forest classifier on the training set, where about 7,600 terms were selected and used for document representation. The classifier was then tested on the test set.

To assess the impact of using captions vs. titles-and-abstracts only, we excuted a second group of experiments, consisting of three sets of experiments over the *GXD-caption* dataset described next. Since this dataset is smaller, we used only five-fold cross validation, employing five complete runs of cross-validation, where a different five-way split of the dataset was used in each complete run, so that each run comprised a total of 25 sessions of training/test, to ensure statistial significance of the results.

For the first set of expriments over the *GXD-caption* set, we used only titles and abstracts of the publications as training/test data. In each of the 25 sessions, we selected about 1,460 *distinguishing* terms based on the training set, which were used as features for representing the documents in the test set.

For the second set of experiments over the *GXD-caption* dataset, we used only image captions of publications in our training/test datasets. We selected about 1,940 *distinguishing* terms based on the training set alone, and used those as features for representing the documents in the test dataset.

In addition to the above experiments, we conducted a third set of experiments over the *GXD-caption* dataset, in which we used text from captions as well as text from titles and abstracts in order to represent documents and classify them. To intergrate the two sources of information into one representation, we first separately identified a set of *distinguishing* terms from the captions, and from the titles and abstracts; we then used the union of the two sets as a basis for representation and classification. The total number of terms used in this integrated representation was about 2,740 per experiment.

As an additional verification step, we also trained a Random Forest classifier using the whole *GXD-caption* dataset (3326 documents) as the training set, where 3438 *distinguishing* terms from title, abstract and captions were selected as features for document representation. We tested the classifier over the set of 1000 *irrelevant* documents selected from among ∼9400 *negative* documents as described in the Methods section.

### Results and analysis

We report results using standard measures widely employed for document classification evaluation, namely *Precision, Recall, F-measure and Accuracy* (45). For biomedical document curation, *Recall* is often viewed as more important than *Precision* because missing relevant documents may compromise the integrity of the database. Therefore, we also include the *utility* measure introduced by TREC Genomics, which biases the evaluation in favor of high *recall*. We use two versions of the *utility* measure: *Utility-10* and *Utility-20*, each giving a different weights to true positives (46). *Utility-10* and *Utility-20* measures are calculated as follows:

$$\mathrm{UTIL}-10=\frac{10\times \mathrm{TP}-\mathrm{FP}}{10\times \left(\mathrm{TP}+\mathrm{FP}\right)},\;\;\mathrm{UTIL}-20=\frac{20\times \mathrm{TP}-\mathrm{FP}}{20\times \left(\mathrm{TP}+\mathrm{FP}\right)}.$$

where *TP* is the number of true positives and *FP* is the number of false positives.

**Figure 1.**
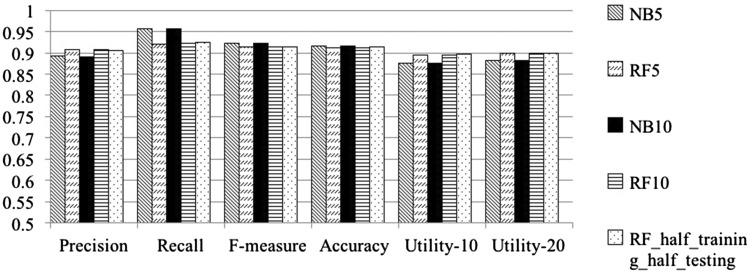
Performance of our classifiers, measured on the GXD dataset according to the different performance metrics, calculated over the various cross-validation settings. NB denotes Naïve Bayes; RF denotes Random Forest classifier. The suffix 5 denotes average over 5 complete runs of 5-fold cross validation (25 runs in total); the suffix 10 denotes average over10 complete runs of 10-fold cross validation (100 runs in total). half-training-halftesting represents runs in which half of the GXD dataset was used for training and the other half for testing.

Table 2 shows the results obtained from the first three sets of cross validation experiments over the *GXD* dataset. Row 1 and Row 2 show results obtained from the Naïve Bayes classifier and from the Random Forest classifier, respectively, where the representation is based on title-and-abstract terms, under 5 complete runs of *5-fold cross validation*. Row 3 and Row 4 show the performance of the two classifiers on the same dataset, under 10 complete runs of *10-fold cross validation*. Row 5 shows results obtained when *half* of the *GXD* dataset was used for training and the other *half* for testing. Figure 1 graphically depicts the results shown in Table 2. Both Table 2 and Figure 1 demonstrate that using our proposed method leads to very high level performance on the large scale *GXD* dataset, according to every evaluation measure, which indicates that the proposed document classification method is effective and can indeed be useful in practice.

**Table 2.** Classification evaluation measures for our classifiers on the *GXD* dataset using different cross-validation settings

Classifiers	Precision	Recall	*F*-measure	Accuracy	Utility-10	Utility-20
NB5	0.892 (0.005)	0.957 (0.003)	0.923 (0.003)	0.917 (0.004)	0.876 (0.006)	0.881 (0.005)
RF5	0.908 (0.006)	0.921 (0.005)	0.915 (0.004)	0.912 (0.005)	0.895 (0.007)	0.899 (0.007)
NB10	0.891 (0.007)	0.957 (0.006)	0.923 (0.004)	0.917 (0.005)	0.875 (0.008)	0.881 (0.008)
RF10	0.908 (0.008)	0.922 (0.008)	0.915 (0.006)	0.912 (0.007)	0.894 (0.009)	0.899 (0.009)
RF-H-and-H	0.905	0.925	0.915	0.913	0.896	0.900

*NB* denotes Naïve Bayes classifier; *RF* denotes Random Forest classifier. The suffix *5* indicates using 5 complete runs of 5-fold cross validation; the suffix *10* indicates using 10 complete runs of 10-fold cross validation. *H-and-H* represents using half of the *GXD* dataset for training and the other half for testing.

**Figure 2.**
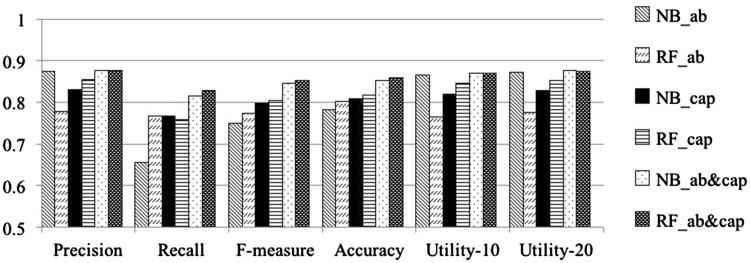
Comparison of classification results obtained over the GXD-caption dataset using different sets of features. NB denotes Naïve Bayes; RF denotes Random Forest classifier. AB indicates using text-features from titles/abstracts only; CAP indicates using features from captions alone; AB_CAP indicates using features from both captions and titles/abstracts. The results shown are averaged over five complete runs of 5-fold cross validation (25 runs in total. Standard deviations are shown in Table 3).

Table 3 shows results obtained from cross validation experiments over the *GXD-caption* dataset. Rows 1 and 2 summarize the performance of the Naïve Bayes and the Random Forest classifiers, respectively, when applied to publications in the *GXD-caption dataset*, represented based on information obtained from title and abstract only. Rows 3 and 4 show the results of the two respective classifiers, when applied to the data represented using terms obtained from the image captions only. Rows 5 and 6 summarize results obtained from the two classifiers when the representation uses terms from *title, abstract* and *image captions*. Table 3 shows that the classifiers generated in the last set of expriments, which rely on features obtained from title, abstract and image captions, have the highest *Precision*, *Recall*, *F-measure*, *Accuracy*, *Utility-10* and *Utility-20.*Figure 2 graphically depicts the results shown in Table 3. In particular, Figure 2 shows that the *Recall, F-measure* and *Accuracy* of our classifiers all clearly increase with the introduction of text from image captions. Both Table 3 and Figure 2 indicate that image captions indeed provide valuable information supporting the GXD document classification task.

**Table 3.** Classification results obtained over the *GXD-caption* dataset using different set of features

Classifiers	Precision	Recall	F-measure	Accuracy	Utility-10	Utility-20
NB_AB	0.874 (0.023)	0.656 (0.027)	0.749 (0.022)	0.783 (0.017)	0.866 (0.024)	0.872 (0.023)
RF_AB	0.779 (0.016)	0.768 (0.024)	0.773 (0.017)	0.802 (0.015)	0.765 (0.018)	0.776 (0.017)
NB_CAP	0.831 (0.018)	0.766 (0.024)	0.797 (0.019)	0.808 (0.017)	0.820 (0.019)	0.828 (0.018)
RF_CAP	0.855 (0.019)	0.758 (0.017)	0.804 (0.015)	0.817 (0.014)	0.846 (0.021)	0.853 (0.020)
NB_AB&CAP	**0.877 (0.018)**	0.816 (0.018)	0.846 (0.014)	0.853 (0.013)	**0.870 (0.019)**	**0.876 (0.018)**
RF_AB&CAP	0.876 (0.019)	**0.829 (0.015)**	**0.852 (0.013)**	**0.858 (0.012)**	0.869 (0.020)	0.875 (0.019)

*AB* indicates using features from titles/abstracts only. *CAP* indicates using features from captions alone. *AB_CAP* indicates using features from both captions and titles/abstracts.

Recall that we also applied our classifier over a set of 1,000 *irrelevant* documents selected as decribed in the Methods section as an additional verification step. The resulting *true negative* rate (the proportion of negative publications that are correctly identified) is 0.863. It is about the same as the *true negative* rate obtained through the cross validation experiments above, which is on average 0.877 with a standard deviation of 0.009. That is, the level of performance obained through the cross validation experiments is retained on documents outside the training/test set. The results thus indicate that our classifiers are indeed effective for addressing the GXD classification task.

## Conclusion and future work

We have presented a biomedical document classification framework for effectively identifying publications relevant to the Mouse Gene Expression Database (GXD) using a realistic large scale dataset. Our *precision, recall, F-measure, accuracy* and *utility* measures are about 90% for all experiments employing different cross validation settings, which shows that our classifier is effective and robust. This performance level is higher than any previously reported over realistically large biomedical document datasets (19, 47), despite the relative simplicity of the classifiers used. We again note that our feature selection step is an important step toward the improved performance. Moreover, our classifier retains a similar level of performance on documents outside the training/test set, which suggests that our classifier is stable and applicable.

Additionally, in terms of efficiency, the total time for pre-processing and classifying a single new publication using our classifier is *20 ms* on average. Thus, our method is sufficiently efficient for supporting the actual document classification task addressed by GXD.

Notably, our experiments use a variety of features obtained from different parts of the publication. Our results indicate that using features obtained from title, abstract and image captions indeed performs best, supporting the idea that the image caption provides substantial evidence for biomedical document classification.

Given the vast amount of *irrelevant* publications (79 284), as part of future work, rather than train a classifier based on a balanced subsample, we plan to further improve the classifier by developing strategies to better utilize more of the *irrelevant* documents as part of the training itself. We also note that as captions were not readily available for all the documents in the *GXD* dataset, the comparative experiments that use caption-based representaiton were limited to the *GXD-caption* dataset, which is smaller. In the future, we plan to use image captions from a larger set of documents, by harvesting figure captions directly from the PDF. We are also actively working on combining information obtained direclty from text as well as the images for addressing biomedical document classification.
