# Effective biomedical document classification for identifying publications relevant to the mouse Gene Expression Database (GXD)

**DOI:** 10.1093/database/baaa043

**Published:** 2020-06-11

**Authors:** Xiangying Jiang, Martin Ringwald, Judith Blake, Haggit Shatkay

**Affiliations:** 1 Department of Computer and Information Sciences, University of Delaware, 101 Smith Hall, Newark, DE, USA; 2 Department of Computer and Information Sciences, The Jackson Laboratory, 600 Main Street, Bar Harbor, ME, USA

The authors thank Dr. Kathleen F. McCoy for pointing out an error in the formulae used for calculating the Utility measures in the original version of the publication “Effective biomedical document classification for identifying publications relevant to the mouse Gene Expression Database (GXD)” by Xiangying Jiang, Martin Ringwald, Judith Blake and Hagit Shatkay (Database, Volume 2017, bax017). As a consequence, two of the tables and two of the figures are also corrected.

In the publication [1], the formulae of utility measures (Page 6, Right Column, Lines 1-2) were provided as:

$$\begin{align*} UTIL-10&=\frac{10\times TP-FP}{10\times(TP+FP)}\\ UTIL-20 &= \frac{20 \times TP-FP}{20\times(TP+FP)}\end{align*}$$

The correct formulae should be as follows (where *FP* in the denominator is replaced by *FN*):

$$\begin{align*} UTIL-10&=\frac{10\times TP-FP}{10\times(TP+FN)}\\ UTIL-20 &= \frac{20 \times TP-FP}{20\times(TP+\textrm{FN})}\end{align*}$$

Accordingly the phrase on Page 6, Right Column, Lines 3-4: “where *TP* is the number of true positives, *FP* is the number of false positives” should be replaced as follows: “where *TP* is the number of true positives, *FP* is the number of false positives, and *FN* denotes false negatives.” 

These errors affect Table 2 and Table 3, as well as Figure 1 and Figure 2.

The rightmost two columns in Table 2 in the original publication [1] were:

**Table:** 

Utility-10	Utility-20
.876(.006)	.881(.005)
.895(.007)	.899(.007)
.875(.008)	.881(.008)
.894(.009)	.899(.009)
.896	.900

The values based on the corrected formulae are:

**Table:** 

Utility-10	Utility-20
.945(.003)	.951(.003)
.912(.005)	.917(.005)
.945(.006)	.951(.006)
.912(.009)	.917(.008)
.916	.921

The complete corrected Table 2 is as follows:

**Table 2:** Classification evaluation measures for our classifiers on the GXD dataset using different cross-validation settings. *NB* denotes Na1ve Bayes classifier; *RF* denotes Random Forest classifier. The suffix *5* indicates using 5 complete runs of 5-fold cross validation; the suffix 10 indicates using *10* complete runs of 10-fold cross validation. *RF-H-and-H* represents using half of the GXD dataset for training and the other half for testing. Standard deviation is shown in parentheses.

Classifiers	Precision	Recall	F-measure	Accuracy	Utility-10	Utility-20
NB5	.892(.005)	.957(.003)	.923(.003)	.917(.004)	.945(.003)	.951(.003)
RF5	.908(.006)	.921(.005)	.915(.004)	.912(.005)	.912(.005)	.917(.005)
NB10	.891(.007)	.957(.006)	.923(.004)	.917(.005)	.945(.006)	.951(.006)
RF10	.908(.008)	.922(.008)	.915(.006)	.912(.007)	.912(.009)	.917(.008)
RF-H-and-H	.905	.925	.915	.913	.916	.921

 Figure 1, which depicts these values showing the performance of our classifiers, was: 

**Figure:**
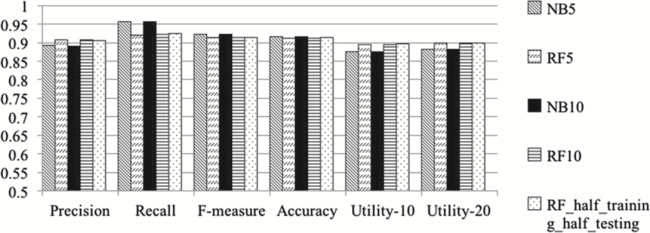


It is updated as follows, where the histograms depicting Utility-10 and Utility-20 have been corrected: 

**Figure:**
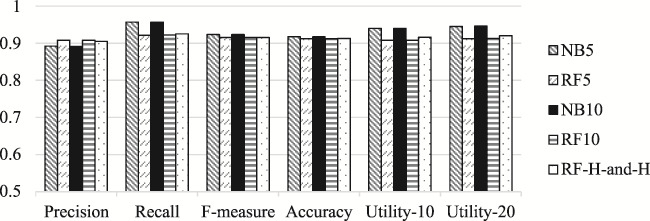


In addition, the rightmost two columns in Table 3 in the original publication were:

**Table:** 

Utility-10	Utility-20
.866(.024)	.872(.023)
.765(.018)	.776(.017)
.820(.019)	.828(.028)
.846(.021)	.853(.020)
**.870(0.19)**	**.876(0.18)**
.869(0.20)	.875(0.19)

The values based on the corrected formulae are:

**Table:** 

Utility-10	Utility-20
.647(.027)	.652(.027)
.746(.024)	.757(.024)
.751(.024)	.758(.024)
.745(.018)	.752(.018)
.805(.018)	.810(.018)
**.817(.015)**	**.823(.015)**

The complete corrected Table 3 is as follows: 

**Table 3:** Classification results obtained over the *GXD-caption* dataset using different set of features. *AB* indicates using features form titles/abstracts only. *CAP* indicates using features from captions alone. *AB&CAP* indicates using features from both captions and titles/abstracts. *NB* denotes Naáve Bayes; *RF* denotes Random Forest classifier. Standard deviation is shown in parentheses. The best performance in each metric is indicated using Boldface.

Classifiers	Precision	Recall	F-measure	Accuracy	Utility-10	Utility-20
NB_AB	.874(.023)	.656(.027)	.749(.022)	.783(.017)	.647(.027)	.652(.027)
RF_AB	.779(.016)	.768(.024)	.773(.017)	.802(.015)	.746(.024)	.757(.024)
NB_CAP	.831(.018)	.766(.024)	.797(.019)	.808(.017)	.751(.024)	.758(.024)
RF_CAP	.855(.019)	.758(.017)	.804(.015)	.817(.014)	.745(.018)	.752(.018)
NB_AB&CAP	**.877(.018)**	.816(.018)	.846(.014)	.853(.013)	.805(.018)	.810(.018)
RF_AB&CAP	.876(.019)	**.829(.015)**	**.852(.013)**	**.858(.012)**	**.817(.015)**	**.823(.015)**

Figure 2, shows a comparison of classification results obtained using the different sets of features: 

**Figure:**
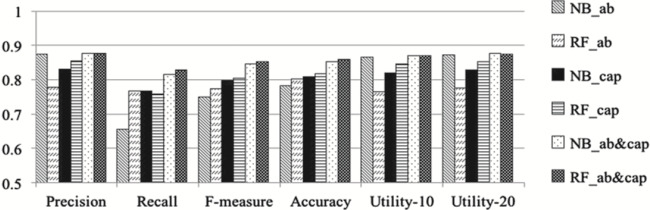


It is updated as follows, where the histograms depicting Utility-10 and Utility-20 have been corrected: 

**Figure:**
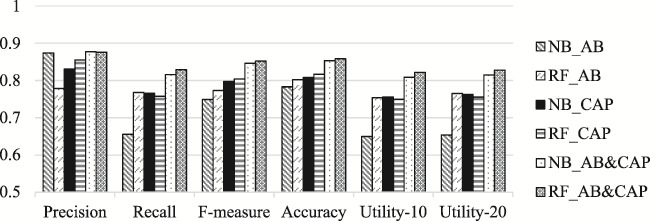


All other parts of the paper including all the conclusions remain unaltered by these corrections. 
